# Supplementary material for: Recombinant Reporter Phage rTUN1::nLuc Enables Rapid Detection and Real-Time Antibiotic Susceptibility Testing of Klebsiella pneumoniae K64 Strains
Source: ACS Sens. 2023 Jan 31;8(2):630–9. doi: 10.1021/acssensors.2c01822 (PMC9972469; doi:10.1021/acssensors.2c01822)
Supplement: Supplementary file 1 — se2c01822_si_001.pdf [file se2c01822_si_001.pdf]

Supporting Information

Recombinant reporter phage rTUN1::*nLuc* enables rapid detection and real-time antibiotic susceptibility testing of *Klebsiella pneumoniae* K64 strains

Peter Braun[a§*], Rene Raab[a§], Joachim J Bugert[a], Simone Braun[a*]

[a]Bundeswehr Institute of Microbiology, 80802, Munich, Germany

*Email: peter3braun@bundeswehr.org
*Email: simoneeckstein@bundeswehr.org

§P.B. and R.R. contributed equally to this paper

Supplementary Figures

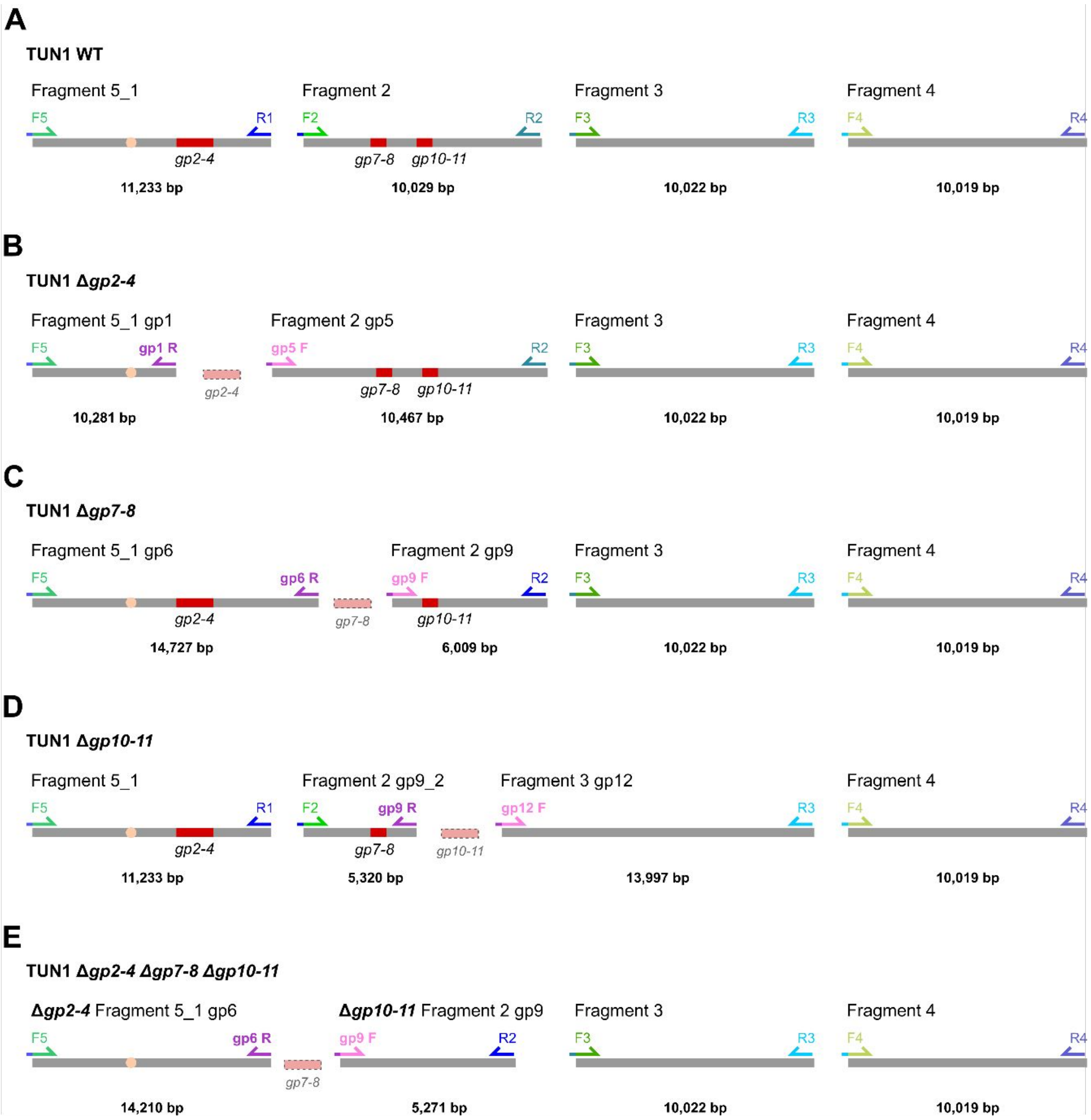

Supplementary Figure S1. Schematic overview of synthetic TUN1 fragments used for *in vitro* DNA assembly. Amplicons used to assemble TUN1 WT (**A**), TUN1 Δ*gp2-4* (**B**), TUN1 Δ*gp7-8* (**C**), TUN1 Δ*gp10-11* (**D**) and TUN1 Δ*gp2-4* Δ*gp7-8* Δ*gp10-11* (**E**) *in vitro*. Gene clusters of interest (for deletion) are marked in red. Deleted gene clusters are marked with a pitched line. One-sided arrows depict primers used for fragment amplification. Orange dots represents genome circularization. If not stated differently, gDNA of TUN1 WT was used as PCR template.

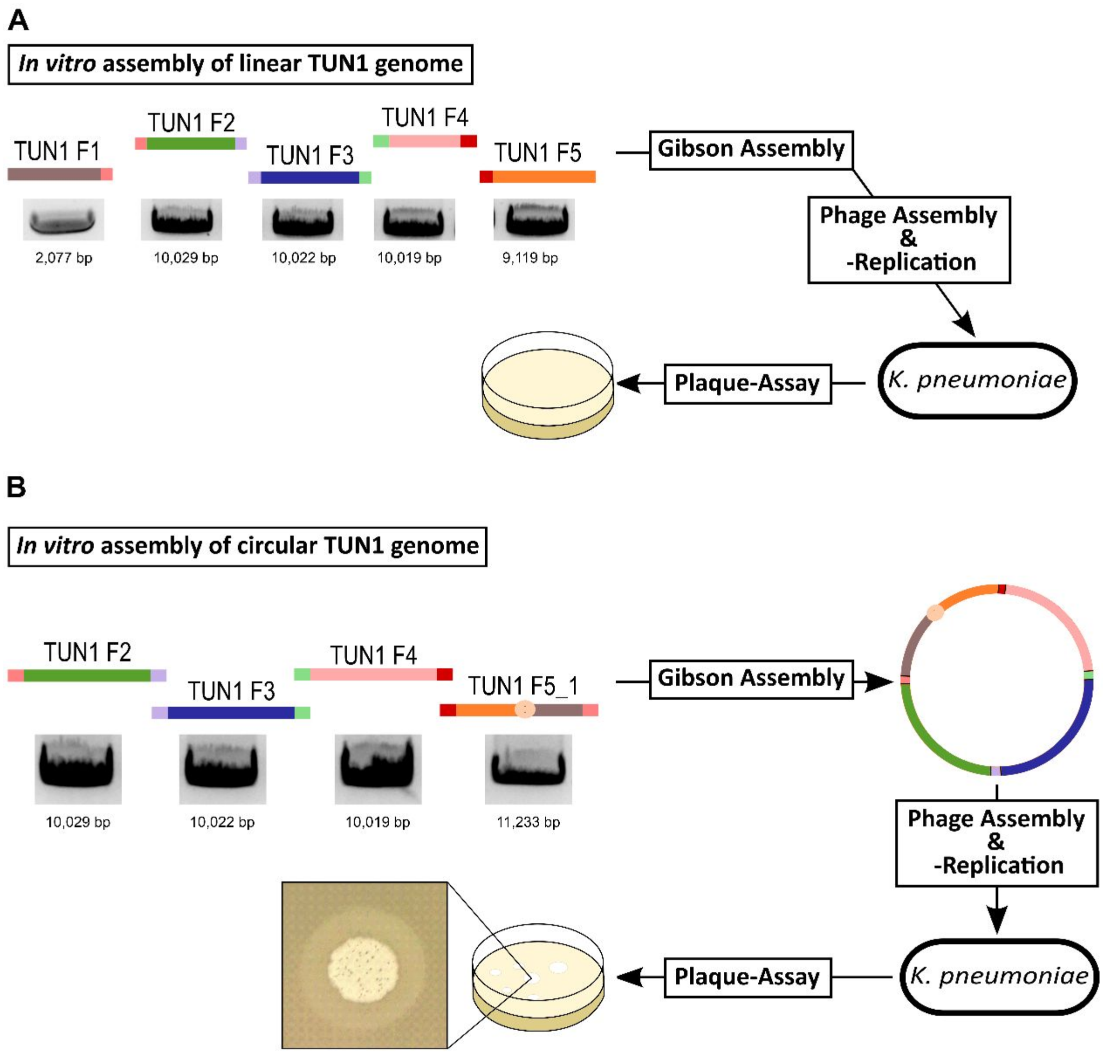

Supplementary Figure S2. Schematic overview of different approached for *in vitro* assembly and rebooting of synthetic TUN1 WT genome. For assembly and rebooting of TUN1 WT all synthetic genome fragments were amplified with overlapping ends between consecutive segments (depicted in matching colors). Subsequently, plaque assays on *Kp* were performed using transformation supernatants. **A)** *In vitro* DNA assembly of linear TUN1 genome. **B)** *In vitro* DNA assembly of circularized TUN1 genome. Plaques, and therefore functional phages, were only obtained when circularized synthetic genomes were used for phage rebooting. The light orange dot depicts the fusion of former Fragment 1 (F1) and Fragment 5 (F5) to Fragment5_1 (F5_1) that enabled circularization of the synthetic genome.

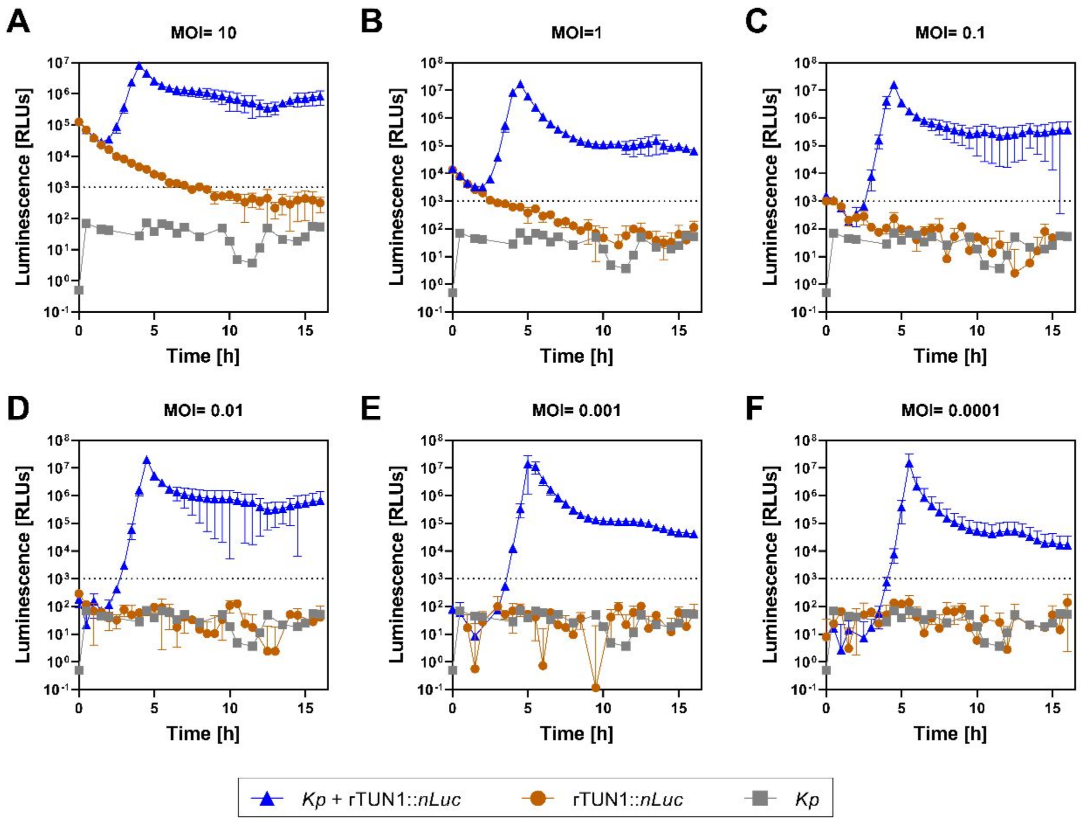

Supplementary Figure S3. Effects of reporter phage titration on luminescence signal. Luminescence signal derived from $1x10^4$ CFUs/well *Kp* 7984 mixed with different rTUN1::*nLuc* concentrations per well (blue triangles): **A)** $1x10^5$ PFUs (MOI= 10) **B)** $1x10^4$ PFUs (MOI= 1) **C)** $1x10^3$ PFUs (MOI= 0.1) **D)** $1x10^2$ PFUs (MOI= 0.01) **E)** $1x10^1$ PFUs (MOI= 0.001) **F)** $1x10^0$ PFUs (MOI= 0.0001)

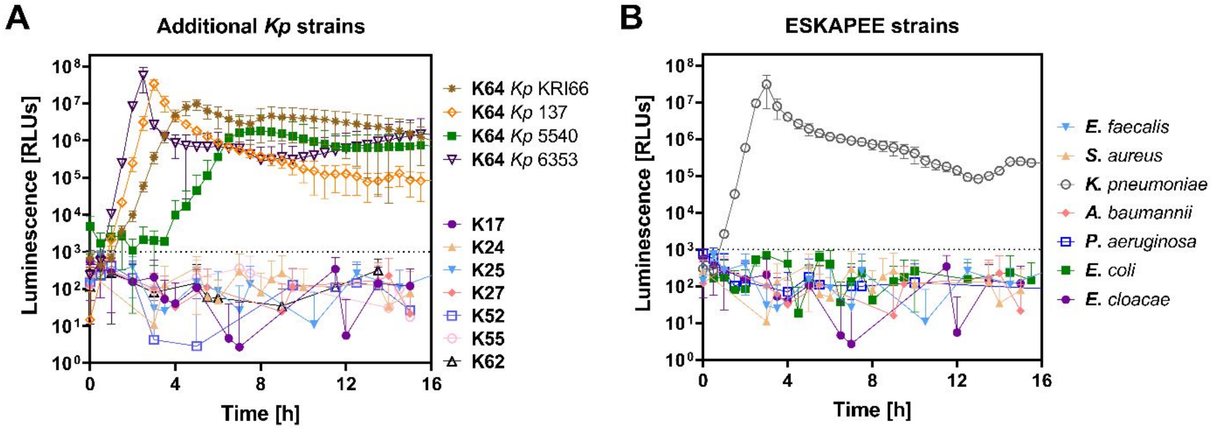

Spplementary Figure S4. rTUN1::*nLuc* derived luminescence on various bacterial strains. Luminescence signal derived from $1x10^2$ PFUs/well rTUN1::*nLuc* mixed with $1x10^6$ CFUs/well of the respective bacterial strain **A)** Luminescence of rTUN1::*nLuc* with *Kp* of different K-types (K64, K17, K24, K25, K27, K52, K55 and K62). **B)** Luminescence of rTUN1::*nLuc* with members of the ESKAPEE group (***E****nterococcus faecalis*, ***S****taphylococcus aureus,* ***K****lebsiella pneumoniae,* ***A****cinetobacter baumannii,* ***P****seudomonas aeruginosa,* ***E****schericia coli,* and ***E****nterobacter cloacae*).

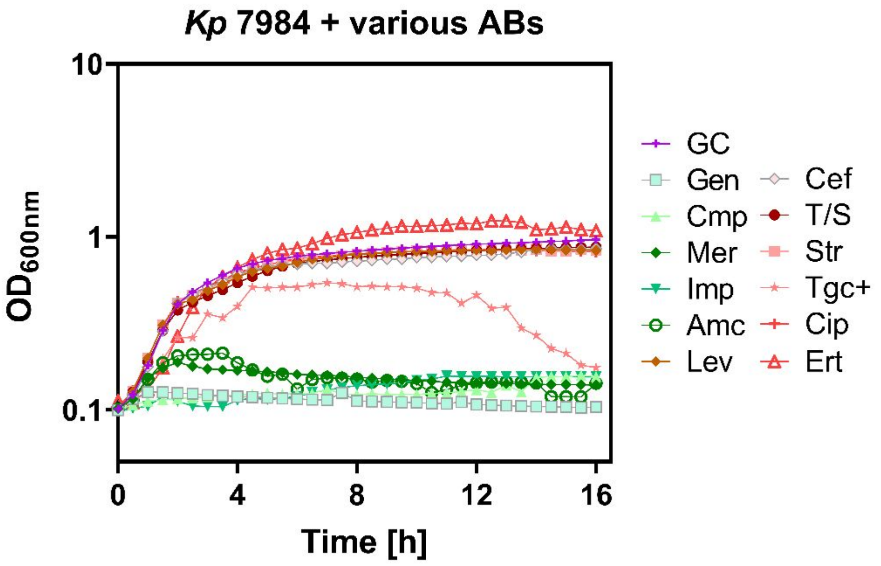

Supplementary Figure S5. Growth curve of *Kp* 7984 with various antibiotics. Growth experiments of *Kp* 7984 revealed that the presence of gentamycin (Gent), chloramphenicol (Cmp), imipenem (Imp), meropenem (Mer) and amoxicillin/clavulanic acid (Amc) inhibited bacterial growth (labeled in green). On the other hand, *Kp* 7984 growth was not affected by the presence of ceftazidim (Caz), streptomycin (Str), tigecyclin (Tgc+), ciprofloxacin (Cip), trimethoprim/sulfamethoxazole (T/S) and ertapenem (Ert) (labeled in red). GC: growth control *Kp* 7984 without AB.

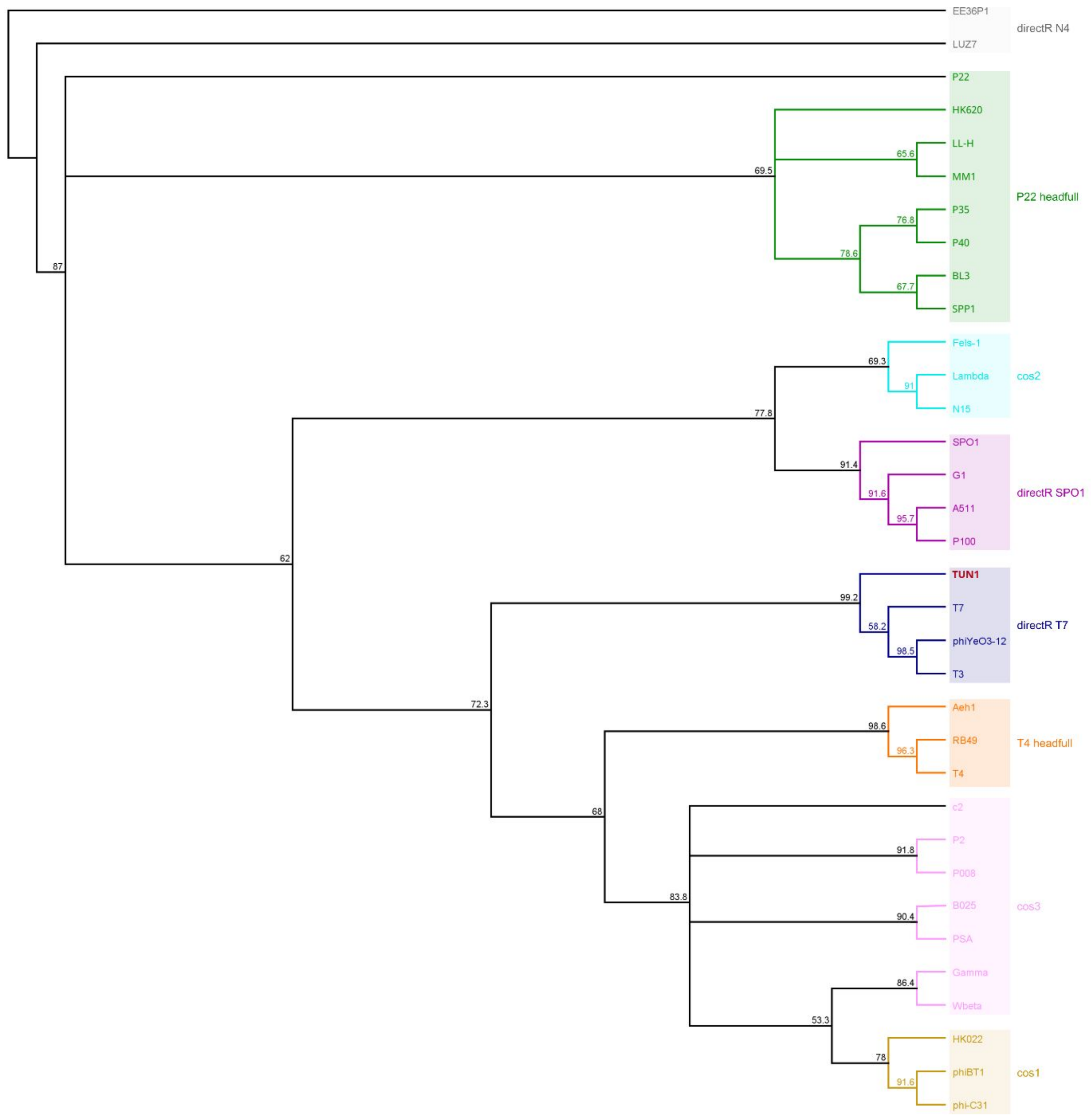

Supplementary Figure S6. Phylogenetic classification of TUN1 Terminase large subunit (TerL). The phylogenetic tree containing TerL sequences of other, well charachterized phages, shows that TerL of TUN1 groups with T7 and therefore, most likely uses the direct repeats T7-like DNA packaging method.

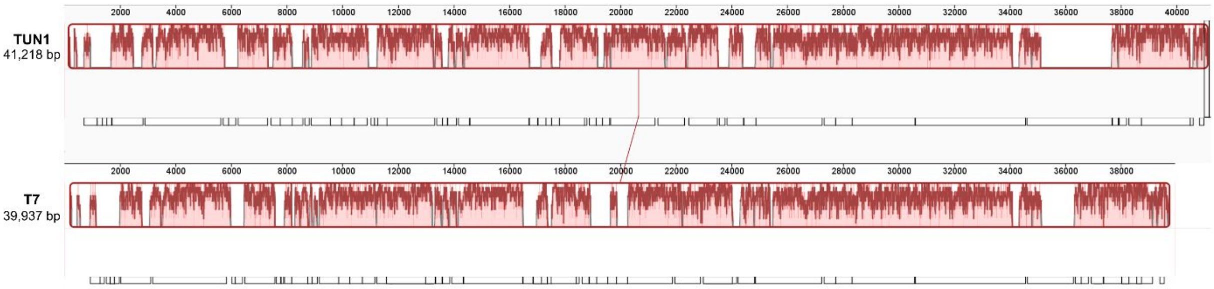

Supplementary Figure S7. Whole genome alignment of phage TUN1 and T7. Comparing the genomes of *Kp* phage TUN1 and *E. coli* phage T7 via whole genome alignment revealed an approx. 45 % pairwise identity. Furthermore, TUN1 harbors additional 1,281 bp compared to T7. The alignment was performed using MAUVE Alignment in Geneious Prime.

Supplementary Tables

Supplementary Table S1. Antibiotics used for susceptibility screening of *Kp* 7984 together with rTUN1::*nLuc.* Minimal inhibition concentrations (MICs) breakpoints referring to antibiotic resistance were adapted from “The European Committee on Antimicrobial Susceptibility Testing, Version 12.0, 2022. http://www.eucast.org”. Breakpoint tables for interpretation of MICs and zone diameters.

Antibiotics	MIC breakpoints R [mg/L]
**Penicillins**	
Amoxicillin/clavulanic acid (uncomplicated UTI only)	> 32 /16
**Chepalosporins**	
Ceftazidime	> 4
**Carbapenems**	
Ertapenem	> 0.5
Imipenem	> 4
Meropenem	> 2
**Fluoroquinolones**	
Moxifloxacin	> 0.25
Ciprofloxacin	> 0.5
Levofloxacin	> 1
**Fluoroquinolones**	
Gentamicin	> 2
Streptomycin	> 1
**Tetracyclines**	
Tigecycline	> 0.5
**Other**	
Trimethoprim/sulfamethoxazol	> 4/76
